# Human B Cells Engage the NCK/PI3K/RAC1 Axis to Internalize Large Particles via the IgM-BCR

**DOI:** 10.3389/fimmu.2019.00415

**Published:** 2019-03-13

**Authors:** Niels J. M. Verstegen, Peter-Paul A. Unger, Julia Z. Walker, Benoit P. Nicolet, Tineke Jorritsma, Jos van Rijssel, Robbert M. Spaapen, Jelle de Wit, Jaap D. van Buul, Anja ten Brinke, S. Marieke van Ham

**Affiliations:** ^1^Department of Immunopathology, Sanquin Research and Landsteiner Laboratory, Amsterdam UMC, University of Amsterdam, Amsterdam, Netherlands; ^2^Synthetic Systems Biology and Nuclear Organization, Swammerdam Institute for Life Sciences, University of Amsterdam, Amsterdam, Netherlands; ^3^Department of Molecular Cell Biology, Sanquin Research and Landsteiner Laboratory, Amsterdam UMC, University of Amsterdam, Amsterdam, Netherlands; ^4^Swammerdam Institute for Life Sciences, University of Amsterdam, Amsterdam, Netherlands

**Keywords:** B cell, CRISPR, internalization, signaling pathway, large antigen-containing particle

## Abstract

Growing evidence indicate that large antigen-containing particles induce potent T cell-dependent high-affinity antibody responses. These responses require large particle internalization after recognition by the B cell receptor (BCR) on B cells. However, the molecular mechanisms governing BCR-mediated internalization remain unclear. Here we use a high-throughput quantitative image analysis approach to discriminate between B cell particle binding and internalization. We systematically show, using small molecule inhibitors, that human B cells require a SYK-dependent IgM-BCR signaling transduction via PI3K to efficiently internalize large anti-IgM-coated particles. IgM-BCR-mediated activation of PI3K involves both the adaptor protein NCK and the co-receptor CD19. Interestingly, we here reveal a strong NCK-dependence without profound requirement of the co-receptor CD19 in B cell responses to large particles. Furthermore, we demonstrate that the IgM-BCR/NCK signaling event facilitates RAC1 activation to promote actin cytoskeleton remodeling necessary for particle engulfment. Thus, we establish NCK/PI3K/RAC1 as an attractive IgM-BCR signaling axis for biological intervention to prevent undesired antibody responses to large particles.

## Introduction

The first step in induction of antibody production is binding of external antigen to the B cell receptor (BCR) on naive B cells. BCR ligation by antigen results in BCR-mediated transmembrane signaling and antigen internalization, followed by proteolytic degradation and presentation of antigen-derived peptides through major histocompatibility complex class II (MHCII) molecules on the B cell plasma membrane (1–3). MHCII/antigen complexes are recognized by antigen-specific T cell receptors expressed by CD4^+^ T cells (4). After formation of a stable antigen-specific interaction, B cells receive help from CD4^+^ T cells via co-stimulatory molecules and soluble cytokines to promote B cell differentiation into high-affinity antibody-producing plasma cells during germinal center (GC) reactions in secondary lymphoid organs.

The BCR consists of a membrane-bound immunoglobulin associated with a CD79a and CD79b heterodimer containing intracellular immunoreceptor tyrosine activation motifs (ITAMs) (5). Upon cognate antigen recognition, phosphorylation of the ITAMs is initiated by the SRC family kinase LYN and spleen tyrosine kinase (SYK) (6–8). These phosphorylated motifs recruit several adaptor and effector proteins that make up the signalosome, containing SYK, B cell linker (BLNK), bruton's tyrosine kinase (BTK), phospholipase C-γ2 (PLCγ2), and the co-receptor CD19. The signalosome drives activation of multiple downstream effector pathways to amplify the signal from the BCR that results in changes in cell metabolism, gene expression, and cytoskeletal organization. Many of proteins required for transmembrane signaling are also involved in antigen internalization and the subsequent intracellular trafficking of the antigen-BCR complex (9). Most studies describing signaling components and molecular mechanisms that control BCR-mediated antigen internalization use small soluble antigens or antigen tethered to planer lipid bilayer surfaces or plasma membrane sheets that is extracted though force-dependent extraction or enzymatic liberation (10–13). B cells are, however, also able to internalize large particles. This ability has long been disregarded, but in recent years, multiple groups, including our own, have demonstrated the existence of this cell biological process for internalization of large particles including anti-IgM-coated bacteria and beads (14–17). The physiological importance of this pathway was recently demonstrated by showing that internalization of large particles by follicular B cells resulted in a strong GC response and the generation of high-affinity class-switched antibodies in mice (16). Of added importance in large particle uptake is the process of epitope spreading. Epitope spreading is a process in which antigens distinct from the antigen that was recognized by the antigen-specific BCR are presented on the B cells plasma membrane (18–20). As such, B cells and CD4^+^ T cells with different specificity can interact to drive the ongoing immune response. This process is highly desirable if it targets foreign antigens during infection to broadening the B cell response. In contrast, in cases of self-reactivity in autoimmune reactions or alloimmunization against transfused blood products, epitope spreading is a clinical problem in much need of targeted therapy.

Here we investigated the molecular mechanisms that mediate internalization and antigen presentation of large particles in human B cells. A high-throughput quantitative image analysis approach was employed using inactivated anti-IgM-coated *Salmonella typhimurium* as a model particle to quantify IgM-BCR-mediated internalization. We show that phosphoinositide-3 kinase (PI3K) is the main driver of actin-dependent large particle acquisition by human B cells. IgM-BCR-mediated activation of PI3K involves both the adaptor protein NCK and the co-receptor CD19 (21–24). We demonstrate that the IgM-BCR/NCK axis is required for internalization of large particles in human B cells. This axis drives internalization via activation of the actin cytoskeleton modulator RAC1. Collectively, our data reveal that the NCK-PI3K-RAC1 axis is essential to mount a humoral immune response to large particles.

## Materials and Methods

### Purification of CD19^+^ B and CD4^+^ T Cells

Human buffy coats were obtained from healthy blood donors after informed consent, in accordance with the protocol of the local institutional review board, the Medical Ethics Committee of Sanquin Blood Supply, and conforms to the principles of the Declaration of Helsinki. Peripheral blood mononuclear cells (PBMCs) were isolated through standard gradient centrifugation using Ficoll-lymphoprep (Axis-Shield). CD19^+^ B cells and CD4^+^ T cells were purified from PBMCs with anti-CD19 and anti-CD4 Dynabeads, respectively, and DETACHaBEAD (Invitrogen) following the manufacturer's instructions. Purity was typically > 98% as assessed by flow cytometry.

### Cell Cultures

HEK293T cells were grown in IMDM (Lonza) supplemented with 10% fetal calf serum (FCS; Bodinco), 100 U/ml penicillin and 100 μg/ml streptomycin (Thermo Fisher Scientific). Ramos B cells were grown in B cell medium that consists of RPMI 1640 medium (Life Technologies) supplemented with 5% FCS, 100 U/ml penicillin and 100 μg/ml streptomycin, 2 mM L-glutamine (Invitrogen), 50 μM β-mercaptoethanol (Sigma) and 20 μg/ml human apotransferrin [Sigma; depleted for human IgG with protein G Sepharose (Amersham Biosciences)]. The HLA-DOβ-GFP Ramos cell line has been described before (17) and was cultured in B cell medium in the presence of 2 mg/ml G418 (Life Technologies).

### gRNA Design and Plasmids

Guide sequences with homology to *CD19* (5′- AAGCGGGGACTCCCGAGACC-3′), *NCK1* (5′-GGTCATAGAGACGTTCCCCT-3′) and *NCK2* (5′-CGGTACATAGCCCGTCCTGT-3′) were designed using CRISPR design, and subsequently cloned into the lentiCRISPRv2 backbone containing puromycin resistance gene (25). The Lifeact-GFP and DORA RAC1-sensor constructs in a lentiviral backbone have been described before (26, 27).

### Lentiviral Vector Construction

Lentiviral vectors were produced by co-transfecting HEK293T cells with the lentiviral transfer plasmids gRNA/Cas9-expressing lentiCRISPRv2, Lifeact-GFP, or DORA RAC1-sensor, and the packaging plasmids pVSVg, psPAX2, and pAdv (28, 29) using polyethylenimine (PEI, Polysciences). Virus-containing supernatant was harvested 48 and 72 h after transfection, then frozen and stored in −80°C.

### Cell Lines and Transduction

Transduction of lentiviral vector into Ramos B cells was performed with 8 μg/ml protamine sulfate (Sigma). CRISPR-mediated knockout cells were enriched by culturing in B cell medium supplemented with 1–2 μg/ml puromycin (Invitrogen). CD19 knockout Ramos B cells were purified using a FACSAria II (BD Bioscience). For this, cells were washed and then stained with anti-CD19 APC (clone SJ25-C1; BD Bioscience) in phosphate buffered saline (PBS; Fresenius Kabi) supplemented with 0.1% bovine serum albumin (BSA; Sigma). The NCK1/2 double-knockout cell line was obtained by single cell sorting using a FACSAria II (BD Bioscience). After clonal expansion, cells were screened for complete knockout using an immunoblot assay (as described below). Ramos B cells that stably expressed Lifeact-GFP or RAC1 biosensor were sorted by flow cytometry-based sorting using a FACSAria II (BD Bioscience).

### Serum Preparation

Blood samples were drawn from healthy volunteers after informed consent (Sanquin). Serum was obtained by collecting blood, allowing it to clot for 1 h at room temperature (RT) and collecting the supernatant after centrifugation at 3,000 rpm for 15 min. Serum of sixteen healthy donors was mixed and stored in small aliquots at −80°C to avoid repetitive freeze/thawing.

### Labeling of Antibodies and Beads

Mouse monoclonal anti-human IgG (MH16-1; Sanquin Reagents), mouse monoclonal anti-human C3d (C3-19; Sanquin Reagents) and mouse monoclonal anti-human IgM (MH15-1, Sanquin Reagents) were labeled with DyLight 650, DyLight 488 or DyLight 405, respectively, according to manufacturer's instructions (Thermo Fisher Scientific). To get rid of excess dye, the antibodies were washed extensively using an Amicon Ultra centrifugal filter (10K; Merck Milipore). The labeling rate was around 7 fluorochromes per antibody, as determined by UV-VIS spectroscopy on a Nanodrop ND1000 spectrophotometer (Thermo Scientific).

Goat-anti-mouse IgG (Fc) polystyrene beads (3 μm, Spherotech) were washed twice with PBS containing 0.1% BSA and labeled overnight with anti-human IgM-DyLight405. The beads were stored at 4°C until further use. Before use, the beads were washed twice with PBS supplemented with 0.1% normal mouse serum (in house), and once with PBS supplemented with 0.1% BSA.

### Bacterial Strains

*Salmonella typhimurium* SL1344 has been described before (30). *S. typhimurium* SL1344 that constitutively express the dsRed protein were generated by electroporating bacteria with a pMW211 plasmid. Bacteria were grown overnight shaking at 37°C in Luria-Bertani (LB) medium broth with 50 μg/ml carbenicillin (Invitrogen). To reach mid-log growth phase, the overnight grown bacteria were diluted 1/33 in fresh LB medium and incubated at 37°C for 3 h while shaking. Subsequently, bacteria were washed twice with PBS and inactivated through incubation at 65°C for 15 min, or through incubation with 4% paraformaldehyde (PFA; Sigma) in PBS for 20 min. To block the free aldehyde groups of PFA, bacteria were incubated with 0.02 M glycine (Merck). Mouse monoclonal anti-human IgM (Fc) (clone MH15-1; Sanquin) or mouse monoclonal anti-human CD19 (clone LT19; Miltenyi Biotec) was mixed with mouse monoclonal anti-*S. typhimurium* LPS (clone 1E6; Biodesign International) and rat anti-mouse IgG1 (clone RM161-1; Sanquin) to generate anti-LPS/IgM (17, 31–33) or anti-LPS/CD19 antibody complexes. Inactivated bacteria were coated with these antibody complexes in the dark for 30 min, while rotating. Subsequently, the bacteria were washed with PBS and kept at 4°C until further use.

For complement/antibody opsonization, *S. typhimurium* was incubated with 10% freshly thawed or heat-inactivated serum in PBS supplemented with 10 mM CaCl_2_ and 2mM MgCl_2_ at 37°C for 30 min. Heat-inactivation of the serum was performed by incubation at 56°C for 30 min. After incubation, bacteria were washed thoroughly with PBS to wash away all non-bound serum components. To asses *S. typhimurium*-reactive antibody and complement opsonization, bacteria were washed and stained with anti-human IgG-DyLight650 and anti-human C3d-DyLight488 in PBS supplemented with 0.1% BSA for 20 min in the dark at RT and measured on a FACSCanto II (BD Bioscience). The acquired data was analyzed using FlowJo Software version 10 (Tree Star).

### Soluble Anti-IgM or Large Particle Challenge

Primary human B cells or Ramos B cells were left untreated or incubated with vehicle (DMSO) or small molecule inhibitors (Supplementary Table 1) in B cell medium without antibiotics for 15 min at 37°C. Subsequently, cells were incubated with soluble anti-IgM (5 μg/mL), uncoated or antibody complex-coated PFA-inactivated *S. typhimurium*, or anti-IgM-coated 3 μm polystyrene beads for 30 min at 37°C. Ice cold PBS was added to halt internalization. Alternatively, B cells were incubated with anti-IgM-coated PFA-inactivated *S. typhimurium* for 30 min in B cell medium without antibiotics on ice to allow particle binding but not internalization (**Figures 3F,G, 5G,H**). Subsequently, the B cells were washed extensively to remove unbound particles, then incubated at 37°C for the time indicated, after which ice cold PBS was added to halt internalization.

### ImageStream^X^ Analysis

Cells were stained with anti-HLA-DR APC (clone L243; BD Bioscience) in PBS supplemented with 0.1% BSA for 30 min in the dark on ice and fixed for 20 min in PBS with 4% PFA. Primary human B cells or Ramos B cells were washed and 4′,6′-diamidino-2-phenylindole (DAPI; Sigma) was added to stain the cell nucleus. Large particle internalization by human B cells was evaluated on an ImageStream^X^ mark II imaging flow cytometer (Merck). The acquired data was analyzed using IDEAS V6.2 Software (Merck) and FlowJo Software version 10 (Supplementary Figures 1A, 4).

### CD4^+^ T Cell Proliferation Assay

Primary human B cells were incubated with vehicle (DMSO) or small molecule inhibitors (Supplementary Table 1) for 15 min at 37°C before being challenged with uncoated (control) or anti-IgM-coated heat-inactivated *S. typhimurium*. Heat-inactivated *S. typhimurium* was used instead of PFA-inactivated *S. typhimurium* to allow antigenic peptide presentation. B cells were primed with *S. typhimurium* for 30 min at 37°C and then washed in medium containing 100 μg/ml gentamicin (Invitrogen) to eliminate non-internalized bacteria (17). To allow antigenic peptide presentation, cells were cultured in B cell medium with 10 μg/ml gentamicin, supplemented with small molecule inhibitors (Supplementary Table 1) for 20 h at 37°C/5% CO_2_. Subsequently, *S. typhimurium-*primed B cells were washed extensively and irradiated with 60 Gy to halt antigen processing before incubation with autologous CD4^+^ T cells that were labeled with Cell Trace CFSE according to manufacturer's instructions (Invitrogen). 10 × 10^4^ B cells and 5 × 10^4^ CD4^+^ T cells were cocultured in 200 μl B cell medium at 37°C/5% CO_2_ in 96-well round-bottom plates (Greiner Bio-One) for 6 days. Cells were then stained with anti-CD4 APC (clone SK3; BD Bioscience) to separate CD4^+^ T cells from the remaining CD19^+^ B cells and CD4^+^ T cell proliferation was measured on a FACSLSRII (BD Bioscience). DAPI was added to exclude dead cells. The acquired data was analyzed using FlowJo Software version 10.

### Lifeact-Imaging

For confocal laser scanning microscopy analysis, Lab-Tec 8-well chamber slides (Thermo Fisher Scientific) were coated with 1 mg/ml poly-L-lysine (Sigma) for 1 h, washed thoroughly with Aquadest and air-dried. Ramos B cells expressing Lifeact-GFP were allowed to bind to the coated slides for 15 min at 37°C before being challenged with anti-human IgM-coated polystyrene beads (3 μm). For real-time imaging a LEICA TCS SP8 confocal microscope system equipped with a 63 × 1.4 NA oil objective and 405 nm diode, 488 nm argon, 594 nm HeNe, and 633 nm HeNe laser, was used. Images were processed using ImageJ software (National Institutes of Health).

### Fluorescence Resonance Energy Transfer (FRET)-Based Biosensor Analysis

RAC1 activity was measured in Ramos B cells after stimulation with anti-IgM-coated *S. typhimurium* by monitoring yellow fluorescent protein (YFP) FRET over donor cyan fluorescent protein (CFP) intensities as described before (27). Lab-Tec 8-well chamber slides were prepared as described above. A Zeiss Observer Z1 microscope equipped with a 63x NA1.3 oil immersion objective, an HXP 120-V excitation light source, a Chroma 510 DCSP dichroic splitter, and two Hamamatsu ORCA-R2 digital charge-coupled device cameras was used for simultaneous monitoring Cerulean3 and Venus emission. Zeiss Zen 2012 microscope software was used to control the system. Offline ratio analyses between Cerulean3 and Venus images were processed using the ImageJ software. Image stacks were background corrected, stacks were aligned, and a smooth filter was applied to both image stacks to improve image quality by reducing noise. FRET ratios were bleed-through corrected (62%) for the CFP leakage into the YFP channel. An image threshold was applied exclusively to the Venus image stack, converting background pixels to “not a number” (NaN) allowing elimination of artifacts in ratio image stemming from the background noise. Finally, the Venus/Cerulean3 ratio was calculated and the Parrot-2 look-up table (created by dr. J. Goedhart) was applied to generate a heatmap.

### Immunoblot Analysis

Ramos B cells (10 × 10^6^) were lysed in 50 mM Tris, pH 7.6, 20 mM MgCl_2_, 150 mM NaCl, 1% (v/v) Triton X-100, 0.5% (w/v) deoxycholic acid (DOC) and 0.1% (w/v) SDS supplemented with a phosphatase inhibitor cocktail (Sigma) and fresh protease-inhibitor-mixture tablets (Roche Applied Science). Cell lysates were then centrifuged at 14,000 rpm for 15 min at 4°C, and supernatants were recovered and boiled in SDS sample buffer containing 4% β-mercaptoethanol. Samples were analyzed using 12.5% SDS-Page. Proteins were transferred onto a 0.2 μm nitrocellulose membrane (Whatman), subsequently membranes were blocked with 5% (w/v) BSA (**Figures 2E, 3K**) or 5% (w/v) milk powder (**Figures 3B, 5G**) in Tris-buffered saline with Tween 20 (TBST). The nitrocellulose membrane was incubated for 1 h at RT with mouse monoclonal anti-NCK (clone 108; BD Bioscience), rabbit polyclonal anti-cofilin (cat #ab42823; Abcam), rabbit polyclonal anti-pAKT (Ser473) (cat #9271; Cell Signaling Technology) or rabbit monoclonal anti-AKT (pan) (clone C67E7; Cell Signaling Technology), followed by incubation for 1 h at RT with HRP-conjugated rat monoclonal anti-mouse kappa (RM-19; Sanquin Reagents) or HRP-conjugated goat anti-rabbit IgG (cat #ab205718; Abcam) in TBST. Between the incubation steps, the membranes were washed with TBST. Antibody staining was visualized with the Pierce enhanced chemiluminescence (ECL) 2 Western Blotting substrate kit (Thermo Fisher Scientific) according to manufacturer's instructions and analyzed using ChemiDoc MP System (BioRad). Brightness/contrast parameters were adjusted globally across the entire image using Image Lab software (BioRad).

### RacGTP Pulldown Assay

Cells were lysed in 50 mM Tris, pH 7.6, 20 mM MgCl_2_, 150 mM NaCl, 1% (v/v) Triton X-100, 0.5% (w/v) DOC and 0.1% (w/v) SDS supplemented with protease inhibitors. Cell lysates were then centrifuged at 14,000 rpm for 15 min at 4°C. Supernatants were recovered and GTP-bound RAC1 was isolated by rotating supernatants at 4°C for 30 min with 5 μg biotinylated PAK-CRIB peptide coupled to streptavidin agarose as described before (27). Beads were centrifuged at 5,000 rpm for 20 s at 4°C, washed five times in 50 mM Tris, pH 7.6, 10 mM MgCl_2_, 150 mM NaCl, 1% (v/v) Triton X-100 and boiled in SDS-sample buffer containing 4% β-mercaptoethanol. Samples were then analyzed by 12.5% SDS-Page as described in the immunoblot section above using mouse monoclonal anti-RAC1 (clone 102; BD Transduction Laboratories) and HRP-conjugated rat monoclonal anti-mouse kappa (RM-19; Sanquin Reagents).

### Statistical Analyses

Statistical analyses were performed using Prism 7 (Graphpad). The statistical tests used are indicated in the figure descriptions. Differences were considered statistically significant when *p* ≤ 0.05.

## Results

### Internalization of Large Particles by Primary Human B Cells Is Mediated via the IgM-B Cell Receptor (BCR) and Not by Antibody and Complement Receptors

**Figure 1:**
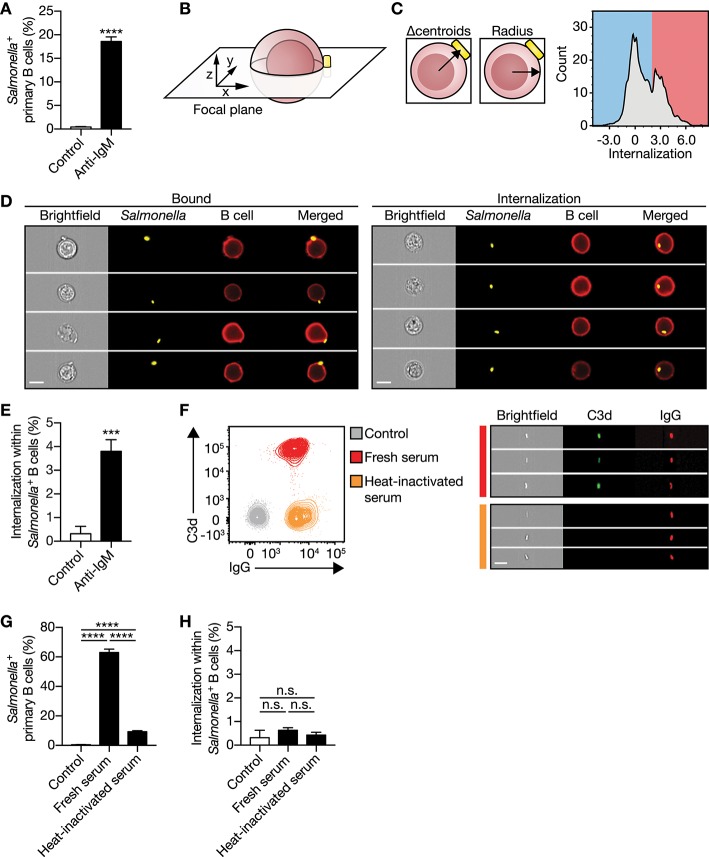
IgM**-**BCR stimulation specifically promotes large particle internalization. **(A)** Proportion of primary human B cells interacting with control or anti-IgM-coated *S. typhimurium* (*n* = 9). **(B)** Schematics of a B cell:*S. typhimurium* interaction. Internalization was assessed using a high-throughput quantitative image analysis approach for *S. typhimurium* located in the same focal plane as the human B cell only. **(C)** To discriminate between bound and internalized *S. typhimurium*, analysis masks were generated to determine the center of both the B cell and *S. typhimurium*. Internalization was defined as the distance between the two centroids (left) after correction for the B cell radius as a measure for the cell size (middle). Internalization was plotted against the event count (right). The red/blue shadings behind the plot indicate the portion of cells that bound (blue) or internalized (red) large particles. Events that had a calculated value similar to or >2 were defined as being internalized. **(D)** Representative images of primary human B cells containing bound (left) or internalized (right) *S. typhimurium*. Bar, 7 μm. **(E)** Proportion of internalization of control or anti-IgM-coated *S. typhimurium* within *Salmonella*^+^ primary human B cells (*n* = 9). **(F)** Representative plots (left) and images (right) of *S. typhimurium* opsonized with antibodies (IgG) or together with complement (C3d) derived from human serum. Bar, 7 μm. **(G–H)** Proportion of primary human B cells that interacted with **(G)** and internalized **(H)** serum-opsonized *S. typhimurium* (*n* = 9). Bars depict mean values and error bars are SEM. ^***^P < 0.001; ^****^P < 0.0001; n.s., not significantly different by paired *t*-test **(A,E)** or repeated-measures one-way ANOVA with Sidak post-test **(G,H)**.

To evaluate internalization of large particles by primary human B cells, a high-throughput quantitative image analyses approach was established using ImageStream^X^, as this combines visual analysis with the statistical power of flow cytometry. Inactivated *S. typhimurium* was used as a model particle. Primary human B cells isolated from blood displayed a low proportion of B cells associating with our model particle (Figure 1A), in line with the low numbers of primary human B cells expressing a BCR that specifically binds *S. typhimurium*, as observed before (17). *S. typhimurium* was coated with a monoclonal antibody specific for immunoglobulin M (anti-IgM) allowing association irrespective of the B cells antigen specificity. Indeed, anti-IgM coating markedly enhanced particle binding as compared with uncoated control (Figure 1A). Subsequently, a custom feature was generated to quantify the relative particle internalization distance into B cells, only taking cells into account in which both particle and B cell were imaged in the same focal plane (Figures 1B,C; Supplementary Figures 1A,B). This allowed us to distinguish between B cells having membrane-bound particles from B cells that completely internalized the particles (Figures 1C,D). Using this approach, we demonstrated that IgM-BCR-mediated internalization of large antigen-coated particles occurred within 30 min of incubation with primary human B cells (Figure 1E), confirming earlier observations (17, 31). To validate this property of the IgM-BCR in large particle internalization, particles were opsonized with serum derived complement and/or antibodies to bind complement- and Fcγ receptors expressed by primary human B cells, respectively (Figure 1F; Supplementary Figure 2). Interestingly, complement and antibody opsonization both mediate particle binding (Figure 1G), but did not facilitate internalization (Figure 1H). Together, these data demonstrate that large particle internalization by human B cells is specifically induced after IgM-BCR-mediated particle binding.

### SYK and PI3K Are Important Signaling Components to Mediate IgM-BCR-Mediated Particle Internalization

**Figure 2:**
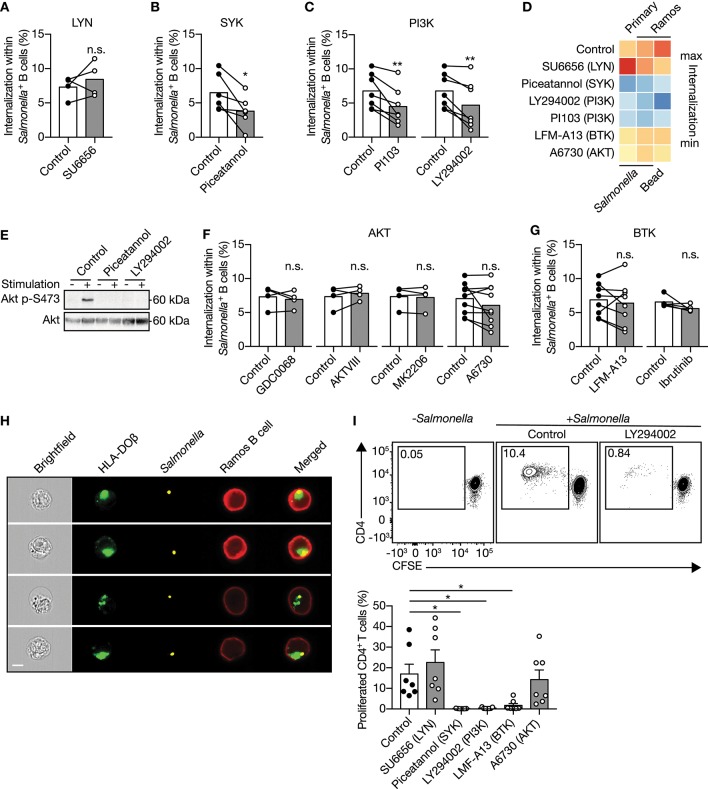
IgM**-**BCR-mediated signaling through SYK and PI3K facilitate large particle internalization. **(A–C)** Proportion of internalization within *S. typhimurium*^+^ primary human B cells after treatment with inhibitors of LYN [**(A)**; *n* = 4], SYK [**(B)**; *n* = 6] or PI3K [**(C)**; *n* = 7]. **(D)** Heatmap of the mean proportion of internalization within *S. typhimurium*^+^ or 3μm polystyrene bead^+^ primary human B cells and Ramos B cells after treatment with inhibitors of LYN, SYK, PI3K, BTK, or AKT. Heatmap colors indicate effect on internalization. **(E)** Immunoblot of whole cell extracts from Ramos B cells that were unstimulated (−) or stimulated (+) with *S. typhimurium* after incubation with inhibitors of SYK (Piceatannol) or PI3K (LY294002). The blots were probed with specific antibodies for pAKT at S473 and AKT. **(F,G)** Proportion of internalization within *S. typhimurium*^+^ primary human B cells after treatment with inhibitors of AKT [**(F)**; *n* = 4 and 9] or BTK [**(G)**; *n* = 8 and 5]. **(H)** Representative images of Ramos B cells expressing GFP-tagged HLA-DOβ having internalized *S. typhimurium. S. typhimurium*-containing phagosomes localize to the HLA-DOβ-containing MHC class II-antigen loading compartments. Bar, 7μm. **(I)** Representative plots (top) and quantification (bottom) of the proportion of proliferated CD4^+^ T cells after co-culture with *S. typhimurium-*primed autologous primary human B cells that were treated with inhibitors (*n* = 7). All data points represent the mean of an individual experiment with duplicate measurements. Error bars indicate SEM. ^*^P < 0.05; ^**^P < 0.01; n.s., not significantly different by paired *t* test **(A–C, F, G)** or repeated-measures one-way ANOVA with Dunnett's post-test **(I)**.

To understand the mechanism by which human B cells facilitate IgM-BCR-mediated internalization of large particles, small molecule inhibitors that decrease activity of signaling proteins downstream of the IgM-BCR were used. Two major players in the signaling cascade downstream of the IgM-BCR include SRC-family protein tyrosine kinase LYN and SYK, which both mediate phosphorylation of conserved ITAMs contained within the cytoplasmic domains of CD79a and CD79b associated with the IgM-BCR (34, 35). The LYN inhibitor SU6656 did not affect internalization by primary human B cells (Figure 2A), whereas the SYK inhibitor piceatannol significantly reduced internalization (Figure 2B), suggesting that large particle uptake is SYK-dependent. A well-known signaling protein that becomes activated after SYK recruitment to the IgM-BCR is PI3K (36). The PI3K inhibitors PI103 and LY294002 significantly reduced internalization of large anti-IgM-coated particles in both primary and Ramos B cells (Figures 2C,D). In line with the fact that PI3K drives AKT phosphorylation (37), inhibition of SYK or PI3K abrogated phosphorylation of AKT at S473 after stimulation with anti-IgM-coated *S. typhimurium* (Figure 2E). In contrast, inhibition of AKT did not affect large particle internalization, indicating that although being activated AKT is not essential for internalization (Figures 2D,F). In addition, inhibition of BTK known to amplify the signal from the IgM-BCR also did not affect large particle internalization, both in primary B cells and in the Ramos B cell line (Figures 2D,G). To assess potential involvement of bacterial virulence factors or pathogen associated molecular patterns (PAMPs) that may engage additional receptors to affect IgM-BCR signaling pathways, inert polystyrene beads were coated with anti-IgM. Anti-IgM-coated polystyrene beads were internalized by Ramos B cells similar to anti-IgM-coated *S. typhimurium* (Figure 2D). Additionally, internalization of polystyrene beads was equally dependent on SYK and PI3K as compared to anti-IgM-coated *S. typhimurium* indicative of a shared mechanism underlying large particle internalization (Figure 2D). Together, these data systematically show that IgM-BCR-mediated uptake of large particles by human B cells is initiated by SYK and requires activation of PI3K irrespective of downstream signaling through AKT or BTK.

### Inhibition of Large Particle Uptake Impairs Presentation to CD4^+^ T Cells

Visualization of internalization of large particles by Ramos B cells revealed that after uptake the particle-containing phagosomes colocalized with HLA-DOβ-containing MHC class II antigen-loading compartments (Figure 2H). To establish whether large particle internalization by primary human B cells led to MHC class II antigen-derived peptide loading and presentation, we assessed their capacity to stimulate autologous CD4^+^ T cells. Particle-primed, but not control primary human B cells induced CD4^+^ T cell proliferation (Figure 2I). Consistent with the effects on large particle internalization, inhibition of SYK and PI3K, in contrast to LYN and AKT in primary human B cells diminished the ability of particle-primed B cells to activate CD4^+^ T cells (Figure 2I). Of note, while BTK inhibition did not affect antigenic particle internalization (Figure 2G), it did significantly abolish CD4^+^ T cells activation (Figure 2I), suggesting that BTK is involved in antigen presentation after large particle internalization.

### Large Particle Induced IgM-BCR Activation of PI3K Strongly Depends on the Adaptor Protein NCK

**Figure 3:**
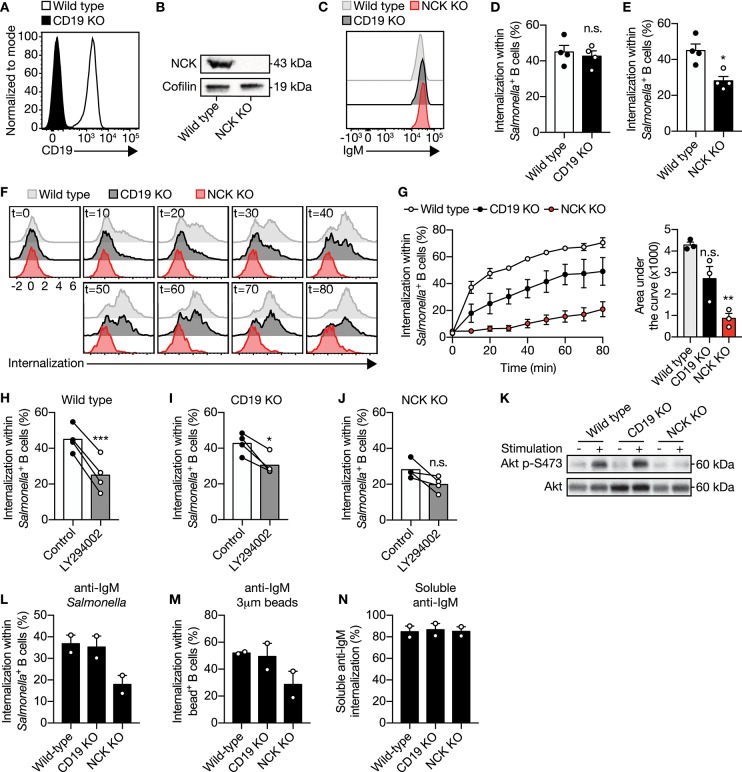
The adaptor protein NCK is required for PI3K activity to facilitate IgM-BCR-induced internalization of large particles. **(A)** Representative histogram of CD19 expression in wild type and CD19 KO Ramos B cells. **(B)** Immunoblot of whole cell extracts from wild type and NCK KO Ramos B cells probed with NCK- or cofilin-specific (loading control) antibodies. **(C)** Representative histogram of surface IgM expression in wild type, CD19 KO and NCK KO Ramos B cells. **(D,E)** Proportion of internalization within *S. typhimurium*^+^ CD19 KO **(D)** and NCK KO **(E)** Ramos B cells compared with wild type (*n* = 4). **(F,G)** Representative histograms **(F)** and quantification [**(G)**; left] of the proportion of internalization within *S. typhimurium*^+^ wild type, CD19 KO and NCK KO Ramos B cells in time (*n* = 3). The area under the curve was obtained to quantify internalization in time [**(G)**; right]. **(H–J)** Proportion of internalization within *S. typhimurium*^+^ wild type **(H)**, CD19 KO **(I)** and NCK KO **(J)** Ramos B cells after treatment with a PI3K inhibitor (*n* = 4). **(K)** Immunoblot of whole cell extracts from wild type, CD19 KO and NCK KO Ramos B cells that were unstimulated (−) or stimulated (+) with *S. typhimurium*. The blots were probed with specific antibodies against pAKT at S473 and AKT. **(L,M)** Proportion of internalization within *S. typhimurium*^+^ **(L)**, 3μm polystyrene bead^+^ **(N)** and soluble anti-IgM^+^ **(M)** wild type, CD19 KO and NCK KO Ramos B cells (*n* = 2). Each data point represents the mean of an individual experiment with duplicate measurements. Error bars indicate SEM. ^*^P < 0.05; ^**^P < 0.01; ^***^P < 0.001; n.s., not significantly different by paired *t*-test **(B, D, G–I)** or repeated-measures one-way ANOVA with Dunnett's post-test **(F)**.

PI3K can be activated through two pathways after IgM-BCR-mediated recognition of antigen. Direct signaling downstream of the IgM-BCR is propagated by the adaptor protein NCK via B cell adaptor for PI3K (BCAP) bound to PI3K (21). Alternatively, the activation of PI3K has been shown to be mediated by CD19 as part of the IgM-BCR co-receptor complex (22–24). To determine which pathway is involved in PI3K-driven uptake of large anti-IgM-coated particles by B cells, CD19 and NCK knockout (KO) Ramos B cells were generated using CRISPR-Cas9 (Figures 3A,B). Plasma membrane IgM expression was not affected upon CD19 or NCK KO (Figure 3C). Targeting of the large particle to the co-receptor CD19 did not induce internalization (Supplementary Figures 3A,B). Remarkably, deletion of NCK and not CD19 significantly decreased IgM-BCR-mediated uptake of large anti-IgM-coated particles as compared to wild type control Ramos B cells (Figures 3D,E). To further assess the role of CD19 and NCK in the dynamics of large particle internalization, internalization of IgM-BCR-bound particles was analyzed in time. In confirmation, absence of NCK strongly and significantly reduced internalization of large anti-IgM-coated particles in Ramos B cells at different time points after particle binding, whereas absence of CD19 did not significantly affect internalization efficiency, although an inhibitory trend was visible (Figures 3F,G). In line with the efficient uptake of large anti-IgM-coated particles in absence of CD19, inhibition of PI3K still significantly decreased internalization in the CD19 KO Ramos B cells, demonstrating a continued dependence on PI3K in the absence of CD19 similar to wild type control (Figures 3H,I). In contrast, inhibition of PI3K along with NCK KO had no significant effect on large anti-IgM-coated particle internalization, suggesting that the adaptor protein NCK is responsible for the recruitment of PI3K into the signalosome to drive large particle internalization (Figure 3J). Indeed, NCK KO, but not CD19 KO Ramos B cells exhibit a lack of phosphorylation of AKT at S473 following stimulation with large particles, which is indicative of diminished PI3K activity (Figure 3K). To determine whether the adaptor protein NCK is required for internalization of large particle in general, the internalization efficiency of anti-IgM-coated *S. typhimurium*, inert anti-IgM-coated polystyrene beads and soluble anti-IgM were compared. Interestingly, absence of NCK strongly reduced internalization of both anti-IgM-coated particles, whereas internalization of soluble anti-IgM was unaffected (Figures 3L–N; Supplementary Figure 4). Altogether, these data demonstrate that uptake of large anti-IgM-coated particles, and not soluble anti-IgM, by the IgM-BCR requires the adaptor protein NCK.

### Actin Polymerization Drives IgM-BCR-Mediated Large Particle Internalization

**Figure 4:**
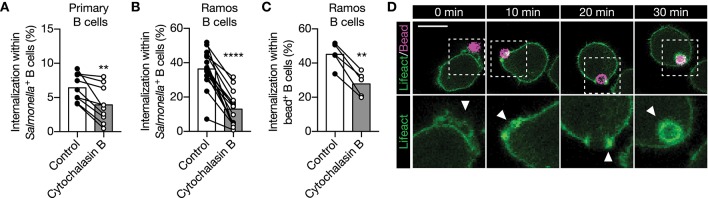
IgM**-**BCR-mediated large particle internalization is an actin-dependent process. **(A–C)** Proportion of internalization within *S. typhimurium*^+^ **(A,B)** or polystyrene bead^+^ **(C)** primary human B cells [**(A)**; *n* = 9], and Ramos B cells [**(B)**; *n* = 16, **(C)**; *n* = 5] after treatment with cytochalisin **(B)**. **(D)** Representative confocal still-images at different time points imaging internalization of anti-IgM-coated polystyrene beads by Ramos B cells expressing Lifeact-GFP. Arrowhead, clusters of actin driving internalization. Bar, 10 μm. The boxed region at the upper panels is enlarged at the lower panels. Each data point represents the mean of an individual experiment with duplicate measurements. ^**^P < 0.01; ^****^P < 0.0001 by paired *t*-test.

We then asked how PI3K induces internalization of large anti-IgM-coated particles. PI3K is a modulator of actin cytoskeleton rearrangement, which is important for BCR mobility and micro cluster formation (12, 38–42). Although disruption of the actin cytoskeleton by cytochalasin B did not alter membrane IgM expression (Supplementary Figures 5A–C), it did significantly decreased internalization of large anti-IgM-coated particles in primary human B cells and in the Ramos B cell line (Figures 4A–C). To further assess the role of the actin cytoskeleton, Ramos B cells expressing Lifeact-GFP were used to effectively visualize actin cytoskeleton remodeling during large particle internalization. Formation of F-actin containing pod-like structure that extends the plasma membrane and surrounds the particle immediately upon contact were visualized using anti-IgM-coated polystyrene beads. F-actin further accumulated during uptake and encircled the particle-containing phagosome long after antigen uptake, which suggests that actin mediates intracellular antigen trafficking (Figure 4D). These data show that BCR-induced internalization of large particles is dependent on actin polymerization.

### NCK Facilitates RAC1 Activity During Internalization of Large Anti-IgM-Coated Particles

**Figure 5:**
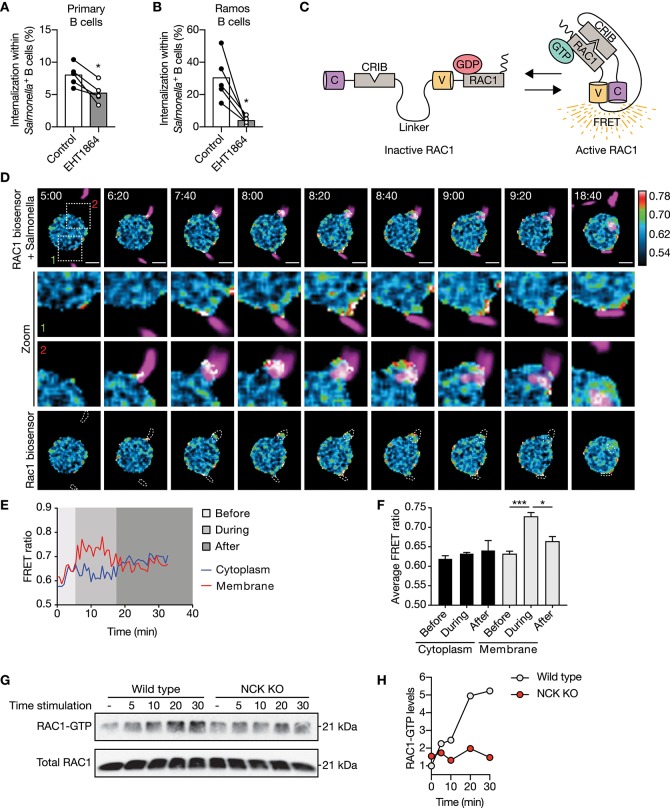
NCK facilitates IgM-BCR-mediated activation of RAC1 subsequent large particle binding. **(A,B)** Proportion of internalization within *S. typhimurium*^+^ primary human B cells **(A)** and Ramos B cells **(B)** after treatment with a RAC1 inhibitor (*n* = 5). **(C)** Schematic illustration of the Cerulean3-CRIB-Venus-RAC1 FRET sensor. Inactive GDP-bound RAC1 results in a large distance between the two fluorescent proteins without a FRET signal. Active GTP-bound RAC1 binds the CDC42/RAC1-interactive binding (CRIB) motif of PAK1, which brings the two fluorescent proteins into close proximity resulting in high FRET efficiency. **(D)** Time-lapse Venus/Cerulean3 ratio images of the RAC1 DORA biosensor showing spatiotemporal RAC1 activation upon stimulation with anti-IgM-coated *S. typhimurium* expressing dsRed (magenta). Bar, 5 μm. The boxed region at the upper panel is enlarged at the middle panels. Lower panels show RAC1 biosensor FRET ratio images with dashed lines marking *S. typhimurium* localization. Time indicated in minutes. Calibration bar shows RAC1 activation (red) relative to basal RAC1 activity (blue). **(E,F)** Representative activation ratio **(E)** and quantification **(F)** of the RAC1 biosensor in time. The activation ratio was assessed at the membrane as compared to the cytoplasm in close proximity to the particle contact area before, during and after *S. typhimurium* internalization (*n* = 3 independent experiments). **(G,H)** Immunoblot and quantification of GTP-bound RAC1 precipitated from whole cell extracts of wild type and NCK KO Ramos B cells that were unstimulated (−) or stimulated with anti-IgM-coated *S. typhimurium* for 5, 10, 20, and 30 min. Total Rac1 levels from whole cell extracts were determined to control precipitation input (representative of *n* = 2 independent experiments). Bars depict mean values and error bars are SEM. Each data point represents the mean of an individual experiment with duplicate measurements. ^*^P < 0.05; ^***^P < 0.001 by paired *t*-test.

We established that NCK/PI3K signaling is required for IgM-BCR-mediated internalization of large particles, which is further propagated irrespective of BTK and AKT. How does PI3K then promote downstream signaling transduction to facilitate actin-dependent internalization? The potential involvement of RAC1 was investigated as RAC1 is a key regulator of the actin cytoskeleton organization in mammalian cells, and its activity is modulated by various guanine nucleotide exchange factors (GEFs) and GTPase-activating proteins (GAPs) that are recruited to phosphorylated lipids produces by PI3K (43–45). Internalization of large anti-IgM-coated particles was significantly affected in both primary and Ramos B cells upon inhibition of RAC1 activity (Figures 5A,B). To further elucidate the role of RAC1 in internalization, a DORA-based RAC1 biosensor was used to visualize RAC1 activity during internalization (Figure 5C). This revealed enhanced RAC1 activity near the particle binding site during particle engulfment, which faded once the particle was fully internalized (Figures 5D–F). In contrast, RAC1 activity in the cytosol was largely unaffected (Figures 5D–F). To establish further a mechanistic link between upstream NCK-dependent PI3K recruitment and downstream RAC1 activity to modulate actin organization, we performed a pull-down assay with the biotinylated CDC42/RAC1-interactive binding (CRIB) domain of PAK1 that binds activated RAC1 from stimulated cells. This analysis validated upregulation of RAC1 activity upon stimulation with large anti-IgM-coated particles in wild type Ramos B cells (Figures 5G,H). In contrast, RAC1 activity was markedly decreased after stimulation of NCK KO Ramos B cells (Figures 5G,H). Together, these data demonstrate a requirement for NCK-mediated signaling in BCR-induced activation of RAC1 during large particle engulfment by human B cells.

## Discussion

BCR engagement with antigen initiates two critical cellular processes in B cells. On the one hand, triggering of the signaling receptor induces B cell activation. On the other hand, antigen encounter promotes internalization and efficient antigen-derived peptide presentation to facilitate an interaction with CD4^+^ T cell help. Over the past decades it has become evident that CD4^+^ T cell help is essential to the development of high affinity, class switched IgG antibody responses (46–52).

**Figure 6:**
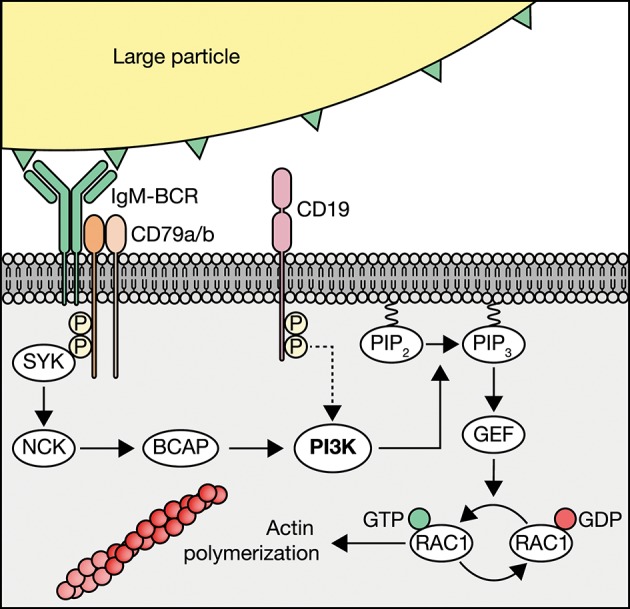
Schematic of IgM-BCR-induced PI3K-driven internalization of large particles. Human B cells internalize large particles though IgM-BCR activation of PI3K via NCK without profound requirement of the co-receptor CD19 (dotted arrow). PI3K facilitates internalization though the conversion of phosphatidylinositol 4,5-biphosphate (PIP_2_) to phosphatidylinositol 3,4,5-triphosphate (PIP_3_) to recruit guanine nucleotide exchange factors (GEFs) that modulate RAC1-dependent activation of the actin cytoskeleton.

In the current study, we aimed to identify the molecular mechanisms that govern internalization of large particles and bacteria by human B cells, as this process enables B cells that recognize one antigen to attract broad T cell help directed against other antigens in the particle, and yields broad undesired antibody responses in autoimmunity and blood transfusion. We present data that demonstrate that human B cells take up large particles via the IgM-BCR-induced NCK/PI3K/RAC1 axis to drive actin cytoskeleton modulation, without a requirement for the co-receptor CD19 (Figure 6). Using our high-throughput quantitative image analysis approach, we established that complement and antibodies opsonization both induce large particle binding, whereas particle internalization was exclusively achieved when the IgM-BCR is engaged. The ability to bind complement and/or antibody-opsonized particles is likely used for antigen transfer. Indeed, non-cognate B cells carry complement-opsonized antigen on their plasma membrane in a CR2-dependent manner to transfer and deposit these antigens onto follicular dendritic cells (FDC) (53). In confirmation with previous observations, we here demonstrate that IgM-BCR-mediated large particle internalization is highly dependent on active transmembrane signaling, which reflects a need for functional intracellular immunoreceptor tyrosine activation motifs (ITAMs) (16, 54). By using small molecule inhibitors, we demonstrate that the signaling protein SYK, and not LYN, is required for large particle internalization. The observation that LYN is not essential in IgM-BCR signaling has been made previously in B cells from *lyn*^−/−^ mice that were found to be hyperresponsive to BCR ligation (55). As strength of the initial BCR signaling correlates with the stability of clustered antigenic receptor molecules, our data suggest that upon proper cross-linking of the IgM-BCR by large particles, signaling can be transmitted independent of LYN, whereas SYK, the protein required for the initiation of the multimolecular signalosome that activates distinct and inter-related signaling pathways, cannot be bypassed (42). BTK inhibition did not affect internalization of large particles, whereas it greatly inhibited particle-dependent CD4^+^ T cell proliferation. This suggests that BTK regulates other intracellular processes that are necessary to mount a proper CD4^+^ T cell response. Indeed, BTK was found to promote the rate of BCR internalization and the movement of the internalized antigen-BCR complex to late endosomes and peptide presentation in splenic mouse B cells (56). This suggests that small soluble antigen and large particles require distinct molecular pathways downstream of the BCR to be internalized, but once internalized similar molecular events are activated, that are dependent on BTK and regulate antigen processing and presentation.

The involvement of the co-receptor CD19 in PI3K recruitment and its central role in BCR signaling are well-known (57, 58). Since PI3K activity was key in the internalization process, the finding that PI3K activity was not profoundly dependent on CD19 was unexpected. It has been observed before that CD19 does not fully account for PI3K translocation to the BCR since PI3K activity is still present in B cells from CD19^−/−^ mice after BCR stimulation (59), as also determined in the current study. Previously, it was shown that BCR signaling in response to small soluble antigen is independent of the co-receptor, whereas CD19 is essential in potentiation BCR-mediated signaling transduction in response to membrane-bound antigen stimulation (42). This may suggest that the co-receptor CD19 is only required for antigen internalization when the antigen is linked to a membrane. Here we demonstrate that PI3K-dependent internalization of large anti-IgM-coated particles is strongly promoted by the adaptor protein NCK, in line with the recent finding that NCK can also propagate BCR-mediated activation of PI3K (21). Although, NCK can be recruited to participate in BCR signaling through the BLNK complex in B cells (60), Castello and colleagues demonstrate that NCK is recruited to a non-ITAM phosphorylated tyrosine on the BCR-associated Igα to participate in BCR signaling in a BTK- and SYK-independent manner. As large particle internalization is dependent on SYK, this may suggest that NCK recruitment is facilitated by the SYK-dependent BLNK route.

It has been described previously that actin cytoskeleton rearrangements are required for internalization of soluble antigen (56). We have extended on this observation by showing that internalization of large particles by human B cells is mediated by the actin cytoskeleton, as also observed for murine follicular B cells (16). Furthermore, we show that actin cytoskeleton rearrangements are modulated by IgM-BCR-induced NCK-PI3K axis via the small GTPase RAC1. PI3K facilitates RAC1 activation through the generation of lipids that can bind and recruit PH-domain-containing guanine nucleotide exchange factors (GEFs) that control RAC1 activation (61). A well-known GEF that might be the bridge between PI3K and RAC1 is VAV, which was shown to activate RAC1 and regulate cytoskeletal structures after BCR activation (34, 62). VAV would be of particular interest since it is the predominant RAC1 GEF expressed in B cells (63) In addition, other PI3K dependent adaptors such as Bam32 have been shown to be an important regulator for RAC1 activation and actin remodeling, warranting future research (64).

In conclusion, we provide evidence for the first time that internalization of large anti-IgM-coated particles by human B cells occurs via the SYK/NCK/PI3K/RAC1-actin axis, which may be susceptible to regulation. SYK and PI3K are both targets of clinically-approved inhibitors that are currently used in clinical trials against autoimmune thrombocytopenia (ITP), leukemia and lymphomas (65, 66). It is important to realize that these patients may be more susceptible to microbial infections due to reduced B cell humoral immune response against microbes as a result of the affected ability to internalize large particles and to attract the CD4^+^ T cell help required for class switching, somatic hypermutation, and plasma cell differentiation.

## Ethics Statement

Approval by local ethical committee (Sanquin Research, Amsterdam) and in line with the Declaration of Helsinki.

## Author Contributions

NV, P-PU, AtB, and SvH conceived the ideas and designed the experiments. NV, P-PU, JW, BN, TJ, and JvR performed the experiments. NV, P-PU, JW, BN, TJ, JvR, RS, JdW, JvB, AtB, and SvH analyzed the data. NV, P-PU, AtB, and SvH wrote the manuscript. All authors have read and approved the manuscript.

### Conflict of Interest Statement

The authors declare that the research was conducted in the absence of any commercial or financial relationships that could be construed as a potential conflict of interest.
