# Supplementary material for: Human B Cells Engage the NCK/PI3K/RAC1 Axis to Internalize Large Particles via the IgM-BCR
Source: Front Immunol. 2019 Mar 13;10:415. doi: 10.3389/fimmu.2019.00415 (PMC6425997; doi:10.3389/fimmu.2019.00415)
Supplement: Supplementary file 1 [file Table_1.DOCX]

| Supplementary Table 1. Small molecule compounds that decrease activity of signaling proteins downstream of the BCR. | | | |
| --- | --- | --- | --- |
| Name | **Inhibitor of** | **Concentration (µM)** | **Manufacturer** |
| SU6656 | LYN | 0.1 | Sigma |
| Pinceatannol | SYK | 0.1 | Sigma |
| LFM-A13 | BTK | 200 | Sigma |
| Ibrutinib | BTK | 1 | Medkoo |
| PI103 | PI3K | 5 | Selleckchem |
| LY294002 | PI3K | 50 | Sigma |
| A6730 | AKT 1/2 | 1 | Sigma |
| MK-2206 | AKT 1/2 | 5 | Selleckchem |
| GDC-0068 | AKT 1/2/3 | 0.5 | Selleckchem |
| VIII | AKT 1/2/3 | 1 | Merck |
| Cytochalasin B | Actin polymerization | 10 | Sigma |
| EHT1864 | RAC1 | 50 | Sigma |
| All inhibitors were titrated and added in a concentration that did not induce cell death of primary human B cells and Ramos B cells. DMSO was used as a negative control. | | | |
