# Supplementary figures and images for: Human B Cells Engage the NCK/PI3K/RAC1 Axis to Internalize Large Particles via the IgM-BCR
Source: Front Immunol. 2019 Mar 13;10:415. doi: 10.3389/fimmu.2019.00415 (PMC6425997; doi:10.3389/fimmu.2019.00415)

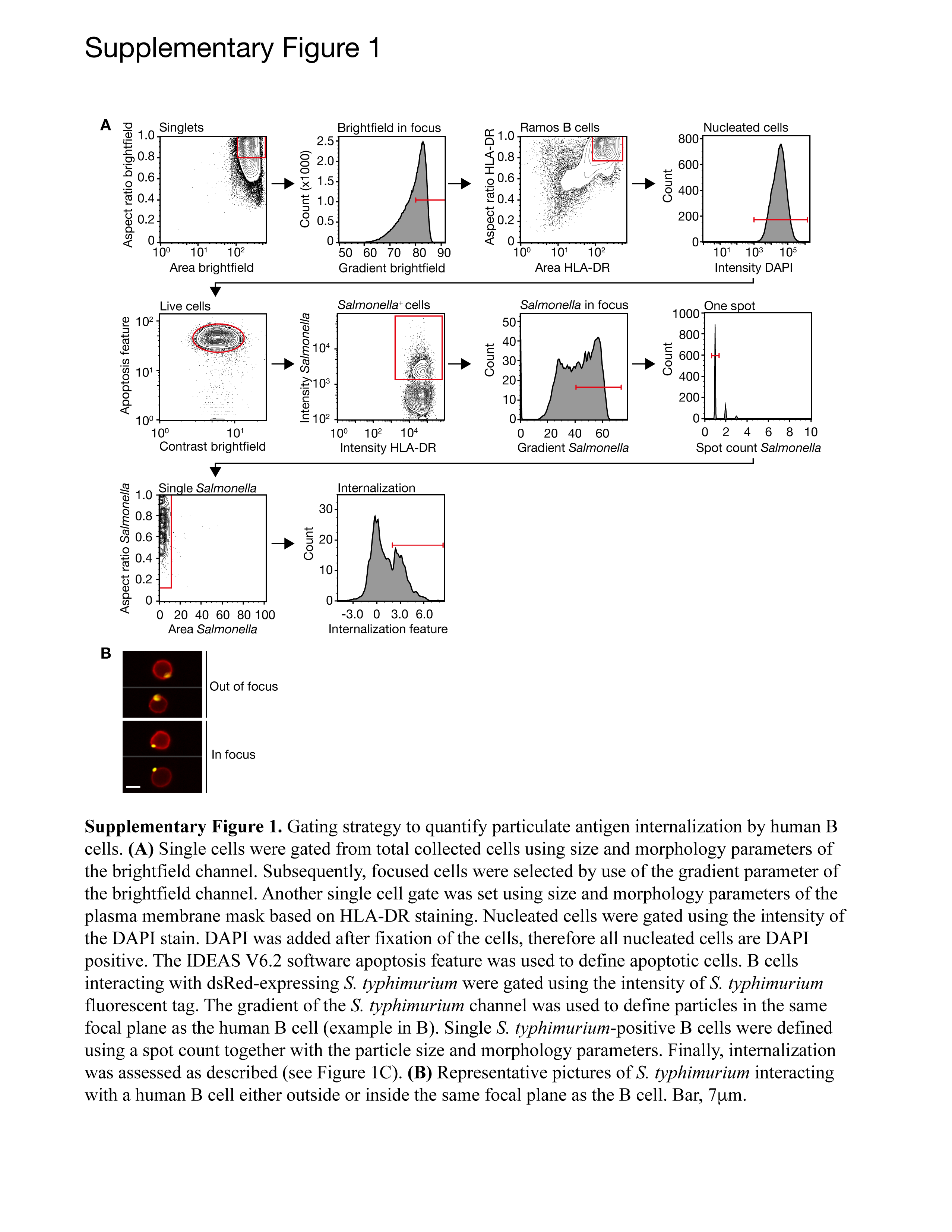

Supplement: Supplementary file 2 [file Image_1.TIF]

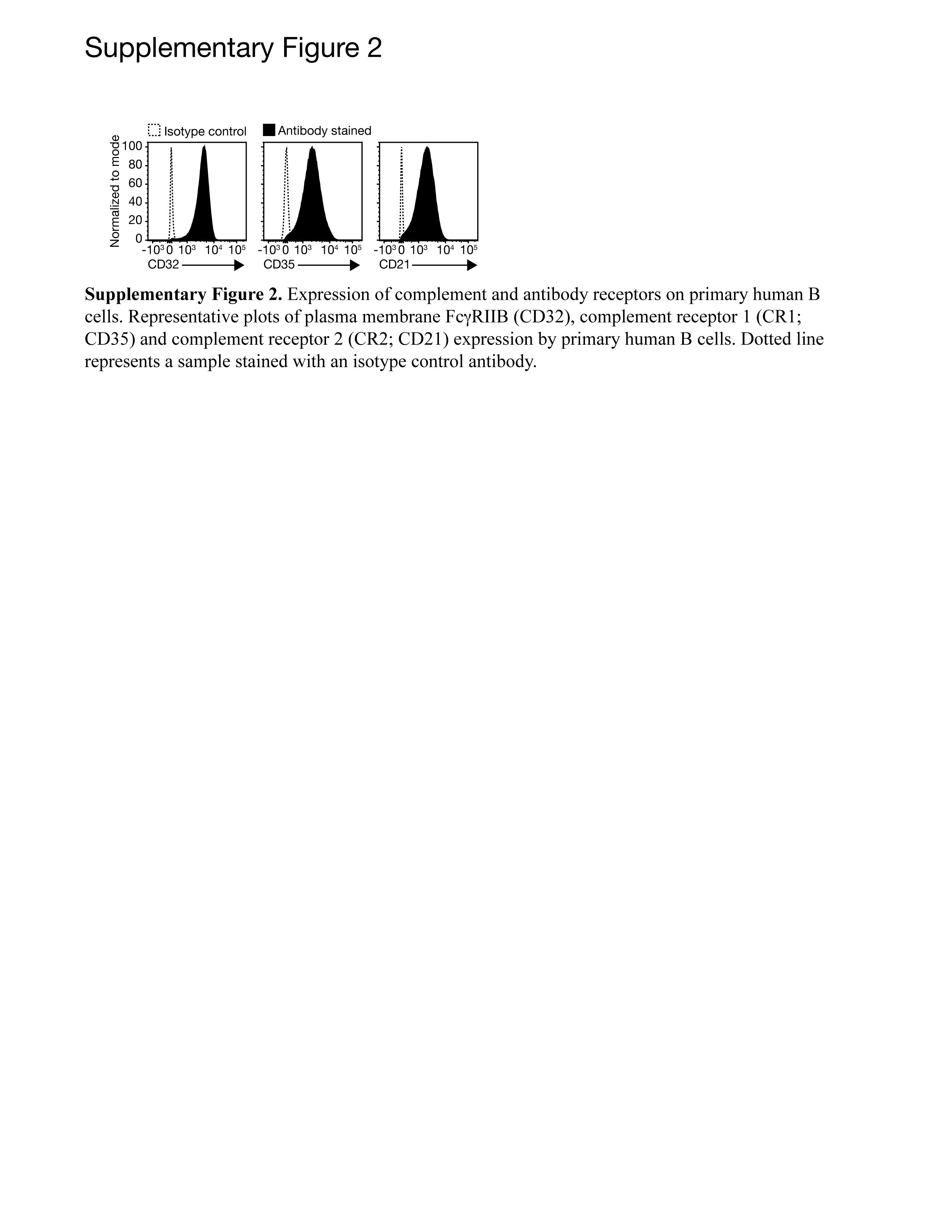

Supplement: Supplementary file 3 [file Image_2.TIF]

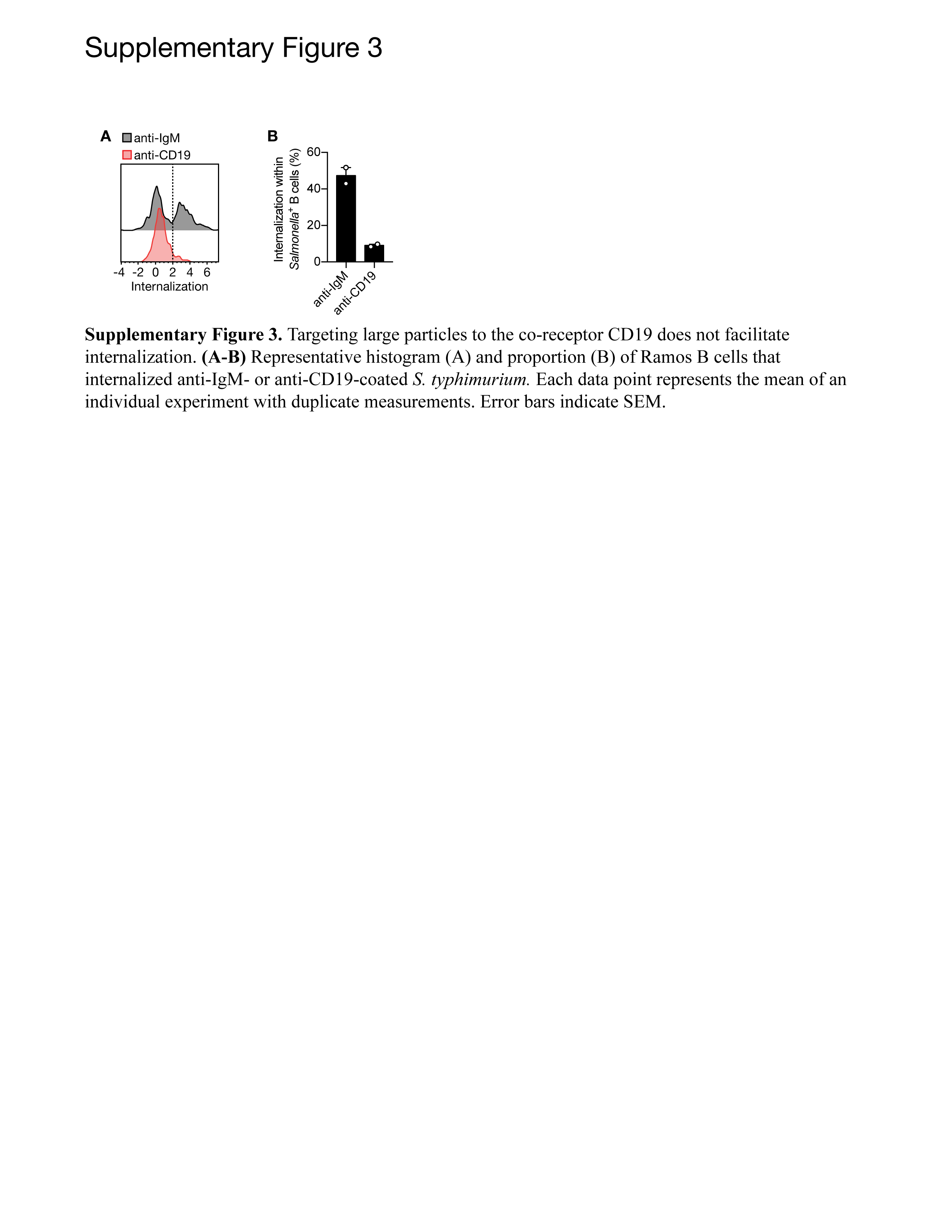

Supplement: Supplementary file 4 [file Image_3.TIF]

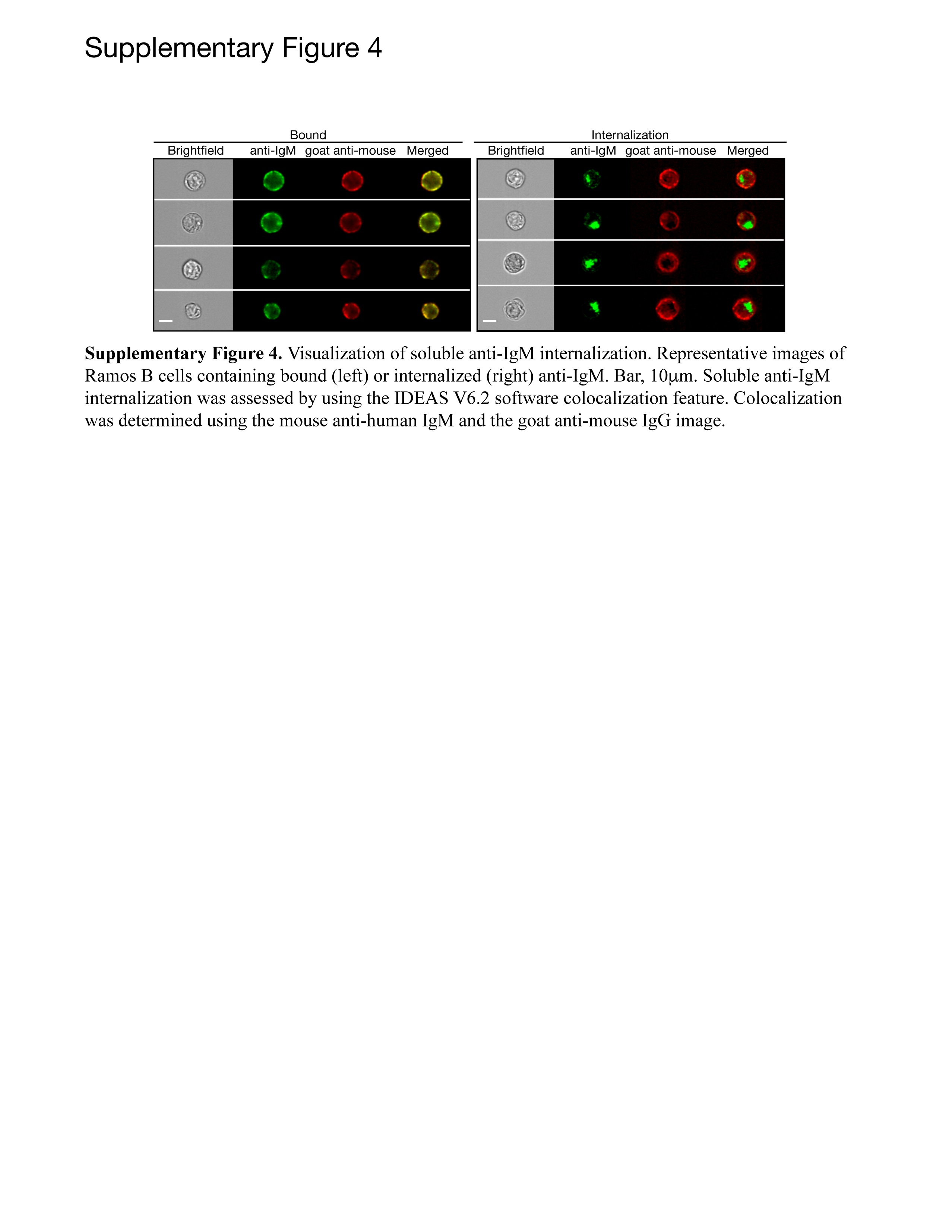

Supplement: Supplementary file 5 [file Image_4.TIF]

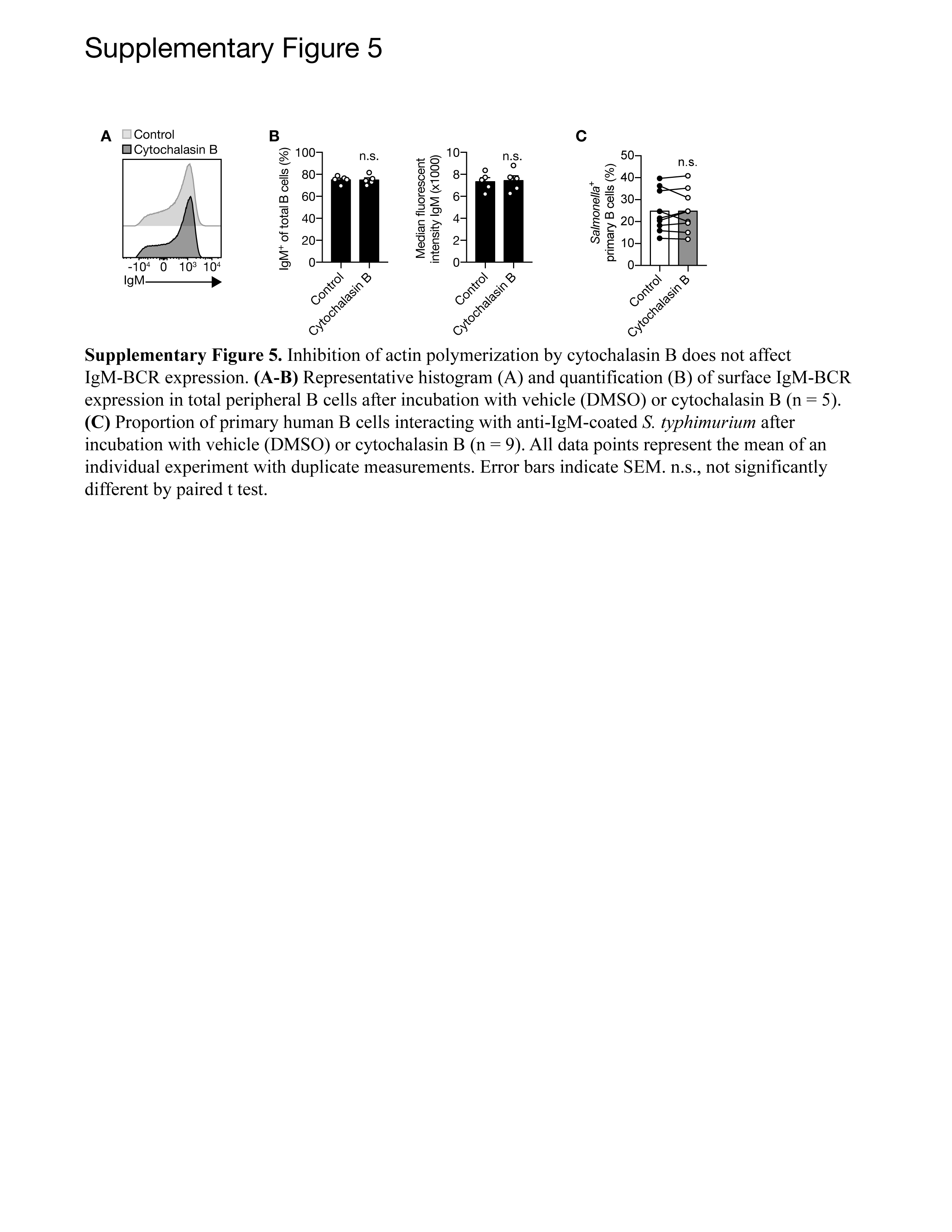

Supplement: Supplementary file 6 [file Image_5.TIF]
